# Pain characteristics and incident frailty in community-dwelling older adults: dose–response relationships and sex differences in a longitudinal study on aging in Taiwan

**DOI:** 10.3389/fpubh.2026.1817545

**Published:** 2026-07-31

**Authors:** Hsiao-Chen Lin, Te-Chia Tseng, En-Chieh Yang, Chih-Jung Yeh, Meng-Chih Lee

**Affiliations:** 1 Department of Public Health, College of Health Care and Management, Chung Shan Medical University, Taichung, Taiwan; 2 Department of Medical Education, Taichung Veterans General Hospital, Taichung, Taiwan; 3 Department of Family Medicine, Taichung Hospital, Ministry of Health and Welfare, Taichung, Taiwan; 4 Institute of Population Health Sciences, National Health Research Institutes, Zhunan Township, Taiwan; 5 College of Management, Chaoyang University of Technology, Taichung, Taiwan; 6 National Taitung University, Taitung, Taiwan

**Keywords:** age, comorbidities, frailty, longitudinal study, pain, prefrailty, sex

## Abstract

**Introduction:**

Frailty, a geriatric syndrome characterized by vulnerability to adverse health outcomes, has become increasingly prevalent with global population aging. Research suggests that pain can predict frailty. However, current evidence is limited by factors such as cross-sectional research designs and specific study populations. In this study, we investigated the associations between pain characteristics (e.g., pain intensity; pain-related interferences in normal work, mood, and sleep; and pain duration) and incident frailty in participants from the Taiwan Longitudinal Study on Aging (TLSA).

**Methods:**

This study included community-dwelling individuals aged 50 and older (*N* = 5,836) without baseline frailty who participated in either of two TLSA survey waves (2011 to 2015 and 2015 to 2019) and were monitored for 4 years. Pain characteristics were assessed using standardized TLSA survey items, and frailty status was determined using a modified Cardiovascular Health Study phenotype. Statistical analyses were performed using covariate-adjusted and unadjusted multinomial logistic regression models. Furthermore, subgroup analyses by age and sex were performed.

**Results:**

Pain characteristics exhibited clear dose–response relationships with incident frailty. Higher pain intensity, greater pain-related interferences in normal work, sleep, and mood; and longer pain duration corresponded to a higher risk of frailty. Pain lasting >6 months and causing moderate to severe interference in normal work increased frailty risk after full adjustment [odds ratio (OR): 1.89 vs. 1.56, *p* < 0.05]. Pain intensity and pain-related interferences in sleep and mood exhibited significantly positive associations with frailty risk in models in which sociodemographic and lifestyle variables were adjusted for (OR: 2.07 vs. 2.17 vs. 2.21, *p* < 0.0001). However, these associations weakened after adjustment for health-related variables. Male participants with moderate to severe pain-related interference in mood had more than twice as high of a frailty risk compared with female participants (OR: 2.22, *p* for interaction = 0.0388).

**Conclusion:**

This study revealed robust dose–response relationships between pain characteristics and incident frailty. Chronic pain and pain-related interference in normal work emerged as strongest predictors of frailty. Significant sex differences were observed. Our findings underscore the importance of comprehensive pain assessment and early intervention in supporting patients at a high risk of frailty.

## 1 Introduction

Life expectancy has increased over the past few decades, but longer life does not equate to better health in old age (1). Frailty has become a key public health challenge with global population aging. Two main concepts of frailty predominate: frailty as a syndrome and frailty as a state (2). Frailty is a geriatric syndrome (3, 4) characterized as a state of increased vulnerability to poor resolution of homeostasis after stress, which results in disproportionate health deterioration due to cumulative loss of physiological reserve (5). Frailty becomes prominent with advancing age and predicts adverse outcomes such as falls, disabilities, hospitalizations, and death (6). A study across more than 60 countries reported that the prevalence of frailty ranged from 11% among community-dwelling individuals aged 50 to 59 years to 51% among those aged ≥90 years (7). Another study analyzing the data of more than 120,000 older adults from 28 countries revealed that the incidence of frailty and that of prefrailty were approximately 43.4 and 150.6 new cases per 1,000 person-years, respectively (8).

Many older adults spend their final years managing the consequences of multiple chronic conditions. Among these conditions, pain is quite common, and its interference with everyday life increases substantially with age (9, 10). Pain is associated with numerous age-related comorbidities; its prevalence can reach 80% among institutionalized individuals (11). Pain contributes to future disability, institutionalization, and increased mortality (12). In the Global Burden of Disease 2021 study, pain—particularly low back pain and headache—was the largest contributor to global disability, posing considerable challenges for countries with aging populations (1).

Because the prevalence of both pain and frailty increases markedly with age, the association between these conditions has garnered considerable attention. Initial data from the Concord Health and Ageing in Men Project highlighted an association between intrusive pain and frailty (13). Shega et al. proposed the concept of “pain homeostenosis” to support the positive association observed between pain and frailty in the Canadian Study of Health and Aging (14). A systematic review indicated that chronic pain can predict frailty, with approximately 45% of all adults with frailty experiencing chronic pain and prevalence reaching 70% (15).

Despite variations in design and measurement, several studies have reported that pain predicts frailty. However, most of these studies were cross-sectional in nature (16–18) or were limited to specific populations (19–22). Given the complex nature of pain (e.g., varied origins, mechanisms, and symptomatology) (23), it has been associated with an impaired functional status, reduced mood (24), poor sleep (25), and increased disability risks (12). According to our review of the literature, no study has compared the risk of frailty across individuals stratified by pain-related interferences in normal work, sleep, or mood. Moreover, few studies have investigated the association between pain intensity and frailty. A longitudinal analysis of data from the English Longitudinal Study of Ageing (ELSA) reported that incident frailty increased with pain severity (mild, moderate, and severe), suggesting a dose–response relationship (26). However, a systematic review concluded that the available evidence was insufficient to determine a dose–response relationship between pain and frailty incidence (11).

In this study, we investigated the associations between pain characteristics and incident frailty from multiple dimensions: (a) pain intensity; (b) pain-related interferences in normal work, mood, and sleep; and (c) pain duration. For this, we analyzed 8-year longitudinal data from a prospective community-dwelling cohort in Taiwan. We further determined whether age and sex moderated the associations between pain characteristics and frailty.

## 2 Methods

### 2.1 Study design and cohort

This population-based longitudinal cohort study was conducted using data from individuals aged ≥50 years who participated in the Taiwan Longitudinal Study on Aging (TLSA). Details of the TLSA can be found elsewhere (27). Trained interviewers conducted face-to-face individual interviews by using structured questionnaires. Data for the period from 2011 to 2019, corresponding to the seventh (2011; *n* = 2,865) and eighth (2015; *n* = 4,101) TLSA survey waves, were analyzed in the present study.

Two generations were included: one was monitored from 2011 to 2015, whereas the other was monitored from 2015 to 2019. The generation monitored from 2011 to 2015 comprised 2,865 individuals. After the removal of participants with missing data on financial situation, frailty score, pain, the Center for Epidemiologic Studies Depression (CES-D) scale score, or health-related variables, the final sample included 2,519 participants. The generation monitored from 2015 to 2019 comprised 4,101 individuals. After the removal of participants with aforementioned missing data, the final sample included 3,715 individuals. A total of 398 participants with frailty at baseline were excluded from the analysis. Thus, the final 4-year follow-up analysis included 5,836 participants.

### 2.2 Measurements

#### 2.2.1 Pain definition

Pain was assessed using the following question: “During the previous 4 weeks, have you experienced any body pain?” Response options were “no pain,” “mild pain,” “moderate pain,” or “severe pain.” On the basis of a prior study (12), responses were categorized into no, mild, and moderate to severe pain for further analysis. The following questions were adapted from the Short Form 12 Health Survey (28):

(a) “During the previous 4 weeks, how much did pain interfere with your normal work (including work outside the home and household work)?”
(b)“During the previous 4 weeks, how much did pain interfere with your sleep?”
(c) “During the previous 4 weeks, how much did pain interfere with your mood?”

The response options for the aforementioned questions were “did not interfere at all,” “mild interference,” “moderate interference,” or “severe interference.” Responses were categorized into no, mild, and moderate to severe interference for further analysis.

(d) “Over the previous 12 months, how long did you experience moderate or severe body pain?”

The response options were “never,” “for <3 months,” “for 3 to 6 months,” “for 7 to 11 months,” or “always felt pain.” Responses were categorized into never, <3 months, 3 to 6 months, or 7 to 11 months or constant pain for further analysis.

#### 2.2.2 Frailty assessment

On the basis of a prior study (29), we defined frailty as per the definition provided by the Cardiovascular Health Study (4). Survey items used in the TLSA were used to define five indicators of frailty:

(a) Unintentional weight loss: measured using the item “Having little appetite and not feeling like eating in the previous week” from the 10-item CES-D scale.
(b) Weakness: measured using the item “Lifting or carrying 20 Taiwanese catties (approximately 12 kg).”
(c) Self-reported exhaustion: measured using the item “Lacking energy to do things in the past week” from the 10-item CES-D scale.
(d) Slow walking speed: measured using the item “Difficulty walking 200 to 300 m.”
(e) Reduced physical activity: measured on the basis of engagement in personal outdoor fitness activities such as gardening, flower arranging, potted plant care, walking, jogging, hiking, and playing ball games.

The response to each item was scored as 0 or 1. Total scores of 0, 1 to 2, and 3 to 5 indicated no frailty, prefrailty, and frailty, respectively. Accordingly, patients were divided into nonfrailty, prefrailty, and frailty groups.

### 2.3 Covariates

#### 2.3.1 Sociodemographic variables

Statistical models were adjusted for the following sociodemographic variables: age, sex, education level, perceived financial situation (“very satisfied,” “satisfied,” “moderate,” “dissatisfied,” or “very dissatisfied”), and marital status (“currently living with a spouse or partner” and “currently living without a spouse or partner”).

#### 2.3.2 Lifestyle variables

Statistical models were adjusted for the following lifestyle variables: current smoking habit (yes or no) and alcohol consumption (yes or no).

#### 2.3.3 Health-related variables

Statistical models were adjusted for the following health-related variables: self-reported physician-diagnosed comorbidities (e.g., hypertension, coronary heart disease, stroke, type 2 diabetes mellitus, renal diseases, asthma, chronic bronchitis, cancer, and arthritis; coded yes or no) and self-rated health (“very good,” “good,” “fair,” “poor,” or “very poor”). The 10-item CES-D scale was used in the TLSA. Maximum and minimum scores for each response were 3 and 0, respectively. A score of ≥10 indicated depressive symptoms.

### 2.4 Statistical analysis

Statistical analyses were performed using SAS (version 9.4; SAS Institute, Cary, NC, USA). Significance level set at *p* < 0.05. The distributions of baseline sociodemographic variables across frailty levels at follow-up were analyzed. Categorical variables were analyzed using the chi-square test and are presented as count (percentage) values. Continuous variables were analyzed using analysis of variance and are presented as mean ± SD values.

The outcome was categorical with three levels (Non-frail, Prefrail, and Frail), so multinomial logistic regression was applied to identify the associations of baseline pain characteristics (2011 and 2015) with frailty at the 4-year follow-up. The results are presented as odds ratios (ORs) with corresponding 95% CIs. Covariates were progressively incorporated during analysis to assess the stability of the associations. The crude model was not adjusted for any covariates. Model 1 was adjusted for age, sex, and baseline frailty status to minimize the confounding effects of demographic characteristics and the initial health status. Model 2 was adjusted for covariates in Model 1 plus perceived financial situation, education level, marital status, smoking habits, and alcohol consumption at baseline to minimize the confounding effects of socioeconomic and lifestyle factors. Model 3 was adjusted for covariates in Model 2 plus self-rated health and comorbidities to minimize the confounding effects of health-related variables. Furthermore, subgroup analyses by age (<65 vs. ≥ 65 y) and sex (male vs. female) were conducted by introducing interaction terms into the logistic regression models to determine potential differences in the associations between pain characteristics and frailty across populations. A *p* value for interaction <0.05 was considered statistically significant.

## 3 Results

Table 1 presents the baseline demographic, life-style and health characteristics of the participants stratified by frailty status at the 4-year follow-up. The study cohort comprised 5,836 individuals, of whom 2,908 (49.82%) were women. The nonfrailty, prefrailty, and frailty groups included 51.19, 41.74, and 7.05% of all participants, respectively. The cumulative incidence of frailty was 7.05%. The proportions of female participants were 44.64 and 8.60% in the prefrailty group and in the frailty group. The proportions of male participants were 38.87 and 5.53% in the prefrailty group and in the frailty group, respectively. The cumulative incidence of frailty among individuals aged <65 years, those aged 65 to 74 years, and those aged ≥75 years were 3.31, 8.52, and 18.68%, respectively. The prevalence of frailty increased with age. Regarding income, higher income satisfaction levels were associated with lower frailty risks. The cumulative incidence of frailty among individuals living with a spouse or partner and those living without a spouse or partner were 6.19 and 9.85%, respectively. Education level was directly correlated with frailty risk, with lower education levels corresponding to higher frailty risks. The cumulative incidence of frailty among individuals with self-rated poor or very poor health and those with self-rated good or very good health were 20.45 and 2.37%, respectively. A higher number of comorbidities was associated with a higher risk of frailty. The baseline demographic, life-style and health characteristics of potentially eligible individuals stratified by inclusion status are summarized in Supplementary Table 1. In total, 1,130 individuals were excluded from our study. Among the excluded individuals, 670 (59.29%) were women, 398 (35.22%) were frail, and 620 (54.87%) were aged ≥75 years. Additionally, excluded individuals had lower income satisfaction, poorer self-rated health, and more comorbidities than did included individuals.

**Table 1:** Baseline demographic, life-style and health characteristics of participants stratified by frailty status (*N* = 5,836).

Variables	Non-frail		Pre-frail		Frail	
	*n*	% (row) or Mean ± SD	*n*	% (row) or Mean ± SD	*n*	% (row) or Mean ± SD
Sex						
Male	1,628	55.60	1,138	38.87	162	5.53
Female	1,360	46.77	1,298	44.64	250	8.60
Age						
<65	1,901	56.23	1,368	40.46	112	3.31
65–74	790	50.61	638	40.87	133	8.52
≥75	297	33.22	430	48.10	167	18.68
Perceived financial situation						
Very dissatisfied, dissatisfied	308	35.94	448	52.28	101	11.79
Moderate	1,038	47.38	993	45.32	160	7.30
Very satisfied, satisfied	1,642	58.90	995	35.69	151	5.42
Spouse						
No	583	41.91	671	48.24	137	9.85
Yes	2,405	54.11	1,765	39.71	275	6.19
Education level						
Illiterate	173	28.83	316	52.67	111	18.50
Primary	978	45.64	976	45.54	189	8.82
Junior/high school	1,173	55.07	878	41.22	79	3.71
College degree or above	664	68.95	266	27.62	33	3.43
Smoking habit						
No	2,554	52.44	1,974	40.53	342	7.02
Yes	434	44.93	462	47.83	70	7.25
Alcohol consumption						
No	1,785	47.64	1,641	43.80	321	8.57
Yes	1,203	57.59	795	38.06	91	4.36
Self-rated health						
Very poor, poor	235	29.49	399	50.06	163	20.45
Fair	1,126	47.27	1,070	44.92	186	7.81
Very good, good	1,627	61.23	967	36.39	63	2.37
Numbers of chronic diseases	2,988	0.88 ± 1.00	2,436	1.12 ± 1.13	412	1.69 ± 1.37

Table 2 provides the baseline pain characteristics of the participants stratified by frailty status at the 4-year follow-up. The cumulative incidence of frailty at follow-up among individuals with no pain at baseline, those with mild pain at baseline, and those with moderate to severe pain at baseline were 5.22, 8.71, and 13.68%, respectively. When participants were stratified by the degree of pain-related interference in normal work, the cumulative incidence of frailty among individuals experiencing no interference, those experiencing mild interference, and those experiencing moderate to severe interference were 5.06, 12.48, and 15.54%, respectively. Similar trends were noted when participants were stratified by the degree of pain-related interference in sleep or mood, with a higher rate of frailty incidence corresponding to a higher degree of pain-related interference. When participants were stratified by the duration of pain over the previous 12 months, the cumulative incidence of frailty among individuals with no pain, those with pain lasting <3 months, those with pain lasting 3 to 6 months, and those with pain lasting 7 to 11 months or constant pain were 5.06, 8.43, 12.65, and 17.75%, respectively.

**Table 2:** Baseline pain characteristics of participants stratified by frailty status at the 4-year follow-up (*N* = 5,836).

Variables	Non-frail		Pre-frail		Frail	
	*n*	%	*n*	%	*n*	%
Pain						
No	2,083	56.34	1,421	38.44	193	5.22
Mild	677	45.71	675	45.58	129	8.71
Moderate to severe	228	34.65	340	51.67	90	13.68
Pain interference of normal work						
No	2,505	55.38	1,789	39.55	229	5.06
Mild	326	41.85	353	45.31	100	12.84
Moderate to severe	157	29.40	294	55.06	83	15.54
Pain interference of sleep						
No	2,649	54.38	1,936	39.75	286	5.87
Mild	208	38.81	267	49.81	61	11.38
Moderate to severe	131	30.54	233	54.31	65	15.15
Pain interference of mood						
No	2,655	54.43	1,942	39.81	281	5.76
Mild	226	36.33	317	50.96	79	12.70
Moderate to severe	107	31.85	177	52.68	52	15.48
Pain in past 12 months						
No	2,239	56.38	1,531	38.55	201	5.06
<3 months	525	45.65	528	45.91	97	8.43
3–6 months	84	33.20	137	54.15	32	12.65
7–11 months or always	140	30.30	240	51.95	82	17.75

Table 3 presents the association of pain characteristics with incident frailty for the participants. Compared with participants experiencing no pain-related interference in normal work, those experiencing mild or moderate to severe interference exhibited progressively elevated risks of frailty (crude model OR: 3.36 vs. 5.79; *p* < 0.0001). The increased risks in individuals experiencing mild interference and those experiencing moderate to severe interference remained significant in Model 3 (OR: 1.47 vs. 1.56; *p* < 0.05). Compared with participants with no pain, those with pain lasting <3 months, 3 to 6 months, or 7 to 11 months or constant pain exhibited progressively elevated risks of both frailty and prefrailty. After adjustment for health-related variables, the longest duration of pain corresponded to the highest risk of frailty. Regarding pain intensity and pain-related interferences in sleep and mood, progressively elevated risks of frailty were observed in the crude model, Model 1, and Model 2. However, in Model 3, the significance of the increased frailty risks decreased after adjustment for comorbidities and self-rated health.

**Table 3:** The association of pain characteristics with incident frailty.

Pain characteristics	Prefrail vs. non-frail						Frail vs. non-frail					
	OR_c_	OR_1_	OR_2_	OR_3_	95% CI (OR_3_)	*p* value (OR_3_)	OR_c_	OR_1_	OR_2_	OR_3_	95% CI (OR_3_)	*p* value (OR_3_)
Pain intensity (vs. no pain)												
Mild pain	1.46^***^	1.27^**^	1.22^**^	1.17	[1.02–1.35]	**0.0250**	2.06^***^	1.59^**^	1.45^**^	1.18	[0.91–1.54]	0.2182
Moderate to severe pain	2.19^***^	1.57^***^	1.41^**^	1.24	[1.01–1.52]	**0.0389**	4.26^***^	2.56^***^	2.07^***^	1.18	[0.84–1.65]	0.3485
Pain interference of normal work (vs. no interference)												
Mild interference	1.52^***^	1.20^*^	1.11	1.03	[0.87–1.24]	0.7133	3.36^***^	2.20^***^	1.89^***^	1.47	[1.09–1.97]	**0.0110**
Moderate to severe interference	2.62^***^	1.78^***^	1.59^***^	1.41	[1.13–1.77]	**0.0029**	5.79^***^	3.18^***^	2.65^***^	1.56	[1.10–2.22]	**0.0122**
Pain interference of sleep (vs. no interference)												
Mild interference	1.76^***^	1.43^**^	1.34^**^	1.26	[1.02–1.55]	**0.0312**	2.72^***^	1.96^***^	1.72^**^	1.32	[0.93–1.87]	0.1201
Moderate to severe interference	2.43^***^	1.68^***^	1.49^**^	1.33	[1.04–1.70]	**0.0255**	4.60^***^	2.70^***^	2.17^***^	1.26	[0.86–1.84]	0.2372
Pain interference of mood (vs. no interference)												
Mild interference	1.92^***^	1.55^***^	1.45^**^	1.32	[1.08–1.61]	**0.0060**	3.30^***^	2.36^***^	2.04^***^	1.43	[1.03–1.98]	**0.0307**
Moderate to severe interference	2.26^***^	1.47^**^	1.34^*^	1.18	[0.90–1.55]	0.2345	4.59^***^	2.61^***^	2.21^***^	1.29	[0.85–1.94]	0.2271
Duration of pain (vs. no pain)												
<3 months	1.47^***^	1.33^**^	1.26^**^	1.20	[1.04–1.40]	**0.0161**	2.06^***^	1.69^**^	1.52^**^	1.21	[0.90–1.61]	0.2046
3–6 months	2.39^***^	1.92^***^	1.72^**^	1.59	[1.18–2.16]	**0.0025**	4.24^***^	2.85^***^	2.33^**^	1.60	[0.98–2.61]	0.0596
7–11 months, always	2.51^***^	1.75^***^	1.61^***^	1.43	[1.13–1.83]	**0.0036**	6.52^***^	3.73^***^	3.16^***^	1.89	[1.32–2.71]	**0.0005**

OR_c_: Crude OR; OR_1_: OR of model 1; OR_2_: OR of model 2; OR_3_: OR of model 3; Model 1 was adjusted for age, sex, and baseline frailty status; Model 2 was adjusted for covariates in Model 1 plus perceived financial situation, education level, marital status, smoking habit, and alcohol consumption at baseline; Model 3 was adjusted for covariates in Model 2 plus comorbidities and self-rated health at baseline; ^***^p < 0.0001, ^**^p < 0.01, ^*^p < 0.05. The bold values mean statistical significance.

Table 4 provides the results of subgroup analyses, respectively, stratified by age and sex after the exclusion of individuals with frailty at baseline. Age exerted no significant moderating effect on the associations between pain characteristics and frailty. However, the risk of frailty was higher in individuals aged ≥65 years than in those aged <65 years. Specifically, mild pain-related interference in sleep, mild pain-related interference in mood, and pain lasting <3 months were associated with prefrailty in older adults and exhibited significant interactions (*p* for interaction <0.05). The risks of frailty and prefrailty varied by sex across pain characteristics. Moderate to severe pain-related interference in mood was significantly associated with frailty in men (OR: 2.22) but not women (*p* for interaction = 0.0388). Conversely, among women, mild pain-related interferences in normal work and mood increased the risk of prefrailty and exhibited significant interactions (*p* for interaction <0.05). Overall, the risk of prefrailty was higher in women than in men across almost all pain characteristics. By contrast, the risk of frailty was higher in men than in women across almost all pain characteristics.

**Table 4:** Results of subgroup analyses by age (≥65 years old and <65 years old) and sex (male and female).

Pain characteristics	Prefrail vs. non-frail			Frail vs. non-frail		
	OR_<65_	OR_≥65_	*p* for interaction	OR_<65_	OR_≥65_	*p* for interaction
Pain intensity (vs. no pain)						
Mild pain	1.06	1.33**	0.0889	1.05	1.31	0.6633
Moderate to severe pain	1.23	1.26	0.7291	1.17	1.12	0.4075
Pain interference of normal work (vs. no pain)						
Mild interference	1.00	1.10	0.5662	1.23	1.64**	0.6389
Moderate to severe interference	1.24	1.65**	0.1347	1.71	1.54	0.3982
Pain interference of sleep (vs. no pain)						
Mild interference	1.03	1.59**	**0.0381**	1.06	1.54	0.5836
Moderate to severe interference	1.38	1.23	0.8493	1.61	0.97	0.0805
Pain interference of mood (vs. no pain)						
Mild interference	1.11	1.63**	**0.0478**	1.45	1.56*	0.8348
Moderate to severe interference	1.24	1.09	0.7981	1.72	0.98	0.0775
Pain of past 12 months (vs. no pain)						
<3 months	1.06	1.45**	**0.0293**	1.08	1.35	0.6901
3–6 months	1.35	2.03**	0.1911	1.22	2.08*	0.5403
7–11 months or always feel pain	1.35	1.60*	0.3689	1.66	1.99**	0.9529

Pain characteristics	Prefrail vs. non-frail			Frail vs. non-frail		
	OR_male_	OR_female_	*p* for interaction	OR_male_	OR_female_	*p* for interaction
Pain intensity (vs. no pain)						
Mild pain	1.20	1.14	0.8000	1.25	1.13	0.9161
Moderate to severe pain	1.14	1.26	0.5545	1.80*	0.81	0.0766
Pain interference of normal work (vs. no pain)						
Mild interference	0.83	1.22	**0.0253**	1.64*	1.45	0.9954
Moderate to severe interference	1.41*	1.40*	0.8994	1.99*	1.25	0.3658
Pain interference of sleep (vs. no pain)						
Mild interference	1.05	1.40*	0.1915	1.18	1.45	0.3878
Moderate to severe interference	1.06	1.52*	0.1803	0.91	1.35	0.2780
Pain interference of mood (vs. no pain)						
Mild interference	1.03	1.59**	**0.0288**	1.39	1.41	0.6377
Moderate to severe interference	1.36	1.09	0.3388	2.22*	0.83	**0.0388**
Pain of past 12 months (vs. no pain)						
<3 months	1.16	1.22	0.6006	1.36	1.15	0.7716
3–6 months	1.21	2.09**	0.0686	1.29	1.96*	0.3317
7–11 months or always feel pain	1.43	1.44*	0.9259	2.16**	1.60	0.6402

Model was adjusted for age or sex, baseline frail status, perceived financial situation, education level, marital status, smoking habit, alcohol consumption, comorbidities, and self-rated health at baseline; ^**^p < 0.01, ^*^p < 0.05. The bold values mean statistical significance.

## 4 Discussion

This longitudinal study revealed associations between pain characteristics and frailty in community-dwelling individuals aged ≥50 years from a representative TLSA cohort. Dose–response relationships were noted across all pain characteristics. Greater pain intensity and pain-related interferences predicted higher frailty risks. Same trends were noted for pain duration, with longer pain episodes corresponding to higher frailty risks. In the comparison of frailty risk across pain characteristics, the highest risk was observed in individuals with pain lasting >6 months or constant pain. The other highest risk was noted in individuals experiencing pain-related interference in normal work, which increased the risk of frailty by 56%. Pain intensity and pain-related interferences in sleep and mood were associated with elevated frailty risks, but these associations became nonsignificant after adjustment for health-related variables. In general, age did not significantly modify the associations between pain characteristics and frailty. However, a sex-specific difference was observed, with the risk of frailty being 2-fold higher in male participants experiencing moderate to severe upset mood due to pain than in their female counterparts.

Frailty at the 4-year follow-up was associated with older age, female sex, low income satisfaction or education level, living without a partner, having a high comorbidity count, poor self-rated health, depressive symptoms, high pain intensity, considerable pain-related interferences with life, and prolonged pain duration. These findings highlight key risk factors for frailty and align with the literature (30, 31). The 4-year incidence rate of frailty was 7.05%, similar to the rate reported by a longitudinal cohort analysis of data from the Cardiovascular Health Study (7.2%) (4).

Pain has been associated with frailty, with risks varying by 1.06 to 3 times across cross-sectional studies (16–18). An analysis of data from the ELSA reported that frailty risk increased with pain severity (26). The risk of frailty was the highest in individuals with the most severe pain (OR: 3.72) (26). Our study revealed a similar upward trend; compared with no pain, moderate to severe pain intensity was associated with a double risk of frailty after adjustment for sociodemographic and lifestyle variables.

Impairment of daily activities is a strong risk factor for frailty (32). Pain was significantly associated with physical frailty. Evidences linked pain to functional limitations and disabilities (e.g., reduced physical activity, slow gait speed, weak grip strength, and functional dependence), which are key components of the frailty phenotype (9, 12, 33). A prospective cohort study from Spain reported similar results; light and moderate to high pain-related interferences with habitual activities elevated the risk of frailty by 30 and 90%, respectively (34). An analysis of 18-year follow-up data revealed that the risk of frailty was 1.71-times higher in individuals experiencing pain-related difficulty standing or walking than in those without pain (35). Pain-related moderate to severe interference in normal work increased the risk of frailty by 64% in a cohort from the Concord Health and Ageing in Men Project (19). Consistent with the aforementioned literature, our study revealed a significant association between pain-related interference in normal work and subsequent frailty risk after adjustment for health-related variables. Mild and moderate to severe pain-related interferences in normal work led to 1.47- and 1.56-fold increases in frailty risk, respectively, compared with the risk noted with no interference.

Chronic pain, defined as pain lasting >3 months, has been associated with multiple adverse health outcomes. It contributes to both the onset and progression of frailty, and the association between these conditions may be attributable to pain homeostenosis (10, 14). Potential mechanisms include physical, physiological, and social factors, such as impaired mobility, reduced metabolism, malnutrition, brain changes (e.g., dysregulated hypothalamic–pituitary–adrenal axis), immune-inflammatory responses, genetic features, and social isolation (e.g., loneliness and depression) (36, 37). A systematic review revealed that persistent pain can double the risk of frailty (11). A study reported that the risk of frailty was 1.85-fold higher in older adults with chronic pain than in those without it after an average follow-up of 5.8 years (38). In the present study, chronic pain lasting >6 months increased the risk of frailty to 1.89 times the risk noted in participants with no pain. Pain duration emerged as the strongest risk factor for frailty. Taken together, these findings highlight the importance of chronic pain diagnosis and suggest that incorporating persistent pain into frailty evaluation can improve the prediction of adverse health outcomes (39).

Poor sleep (40) and upset mood (41) were associated with frailty. In the present study, frailty exhibited dose–response relationships with poor sleep and upset mood due to pain, but the associations weakened after adjustment for comorbidities and self-rated health. Frailty is independent of comorbidities, but their overlap is common and increases with increasing frailty (5, 42). Comorbidities and self-rated health (43) are predictors of frailty. Notably, a high comorbidity count is independently associated with chronic pain (44), and individuals often rate overall health as poor while experiencing pain (12). Therefore, the weakened associations reflect the concomitant and highly interrelated nature of pain-related symptoms (sleep and mood) and health-related variables (comorbidities and self-rated health). Adjustment for health-related variables might have controlled for the common mechanisms they share, thereby weakening the associations between pain characteristics and frailty. Rodrigues et al. reported a similar phenomenon in a prospective cohort, where ORs decreased and lost statistical significance after adjustment for pain-related chronic conditions (34). In the present study, the effects of pain on sleep and mood increased the risk of frailty. Further studies are required to elucidate the intricate interactions of pain with sleep and mood and identify effective strategies for mitigating the effects of these interactions on health and wellbeing.

Evidence suggests that pain is higher among women than among men (31). Female sex is independently associated with chronic pain in geriatric outpatients (45). Notably, an analysis of data from the ELSA revealed no significant effect of sex on the association between pain and frailty (26). An explanation for the observations may be that women tend to report more pain symptoms than do men because of different social learning experiences (46). Furthermore, women tend to have relatively frequent health-care utilization, which enables them to receive medical knowledge and support; this expands their social networks and resources, thereby enhancing resilience and preventing adverse health outcomes such as frailty (21, 47). Our study revealed one association between pain characteristics and frailty significantly modified by sex. The one was that men who experienced severe pain-related upset mood had double the risk of frailty than did women. The literature may explain the sex-based difference observed in our study. Affleck et al. reported that the “carry-over” effect of pain on mood over the following days is stronger among men than among women, whereas the use of coping strategies for preventing emotional sequela is higher among women than among men (46). Although most associations between pain characteristics and frailty were not significantly affected by sex and age in the present study, several sex- and age-specific risk factors for prefrailty warrant attention. Pain-related interference in sleep or mood increased the risk of prefrailty (*p* for interaction <0.05) in individuals aged >65 years. Sex-based differences were evident. The risk of prefrailty was higher in women than in men when pain interfered with normal work and mood. Further studies are required to elucidate age- and sex-based differences across populations with distinct pain characteristics and the effects of these modifiers on the risks of frailty and prefrailty. Insights from such studies may inform strategies for early prevention of adverse sequela.

Our findings indicated that pain characteristics were associated with increased risks of frailty and prefrailty. These findings corroborate evidence underscoring high pain-related interference in daily life (48, 49), prolonged pain duration (15, 48, 49), and intense pain (15, 48) as strong generic prognostic factors in primary pain care. Therefore, assessing pain characteristics may help physicians accurately identify geriatric patients at elevated risks of frailty. Frailty is a dynamic and potentially reversible condition; individuals may transition from robust health to prefrailty or from prefrailty to frailty over time, but most people progress toward higher frailty levels (5, 47). Therefore, early and effective pain management is crucial for preventing prefrailty and thus frailty (50). Our analysis might have underestimated the true relationship between pain and frailty because we excluded individuals who were already frail or in very poor health at baseline. Our cohort comprised younger participants (≥50 y), which enhanced the generalizability of the findings to the broader population and reflected real-world demands of contemporary health care.

This study has several strengths. First, to the best of our knowledge, this study is the first to explore the relationship between pain and frailty in a TLSA cohort and to investigate the associations between pain characteristics and frailty. Our findings suggest that pain influences the incidence of frailty through multiple dimensions. Second, this study was conducted using a TLSA cohort, a representative population of community-dwelling adults in Taiwan. Third, the measurement tools used have been validated previously (29, 40). Finally, individuals with frailty at baseline were excluded from the analysis to focus on incident frailty.

Our study has some limitations. We analyzed data that were collected using a self-report questionnaire, which might have introduced recall bias. Moreover, we defined frailty status on the basis of a modified Fried physical frailty phenotype. Given that different tools may lead to different results, further research comparing this approach with other tools, such as frailty index based on different deficit compositions, is required. However, the aforementioned frailty phenotype predicts adverse health outcomes such as mortality and poor quality of life (2). Thus, our findings remain credible and robust. We performed multiple interaction tests (e.g., by sex and age), which increased the likelihood of borderline significant *p* values and inflated false discovery rates. Additionally, the inclusion of individuals with baseline prefrailty may have introduced confounding bias. Therefore, the observed sex- and age-specific differences should be interpreted with caution. Nonetheless, the purpose of the analysis was to draw attention to the topic and encourage further research in this area.

## 5 Conclusion

The present study revealed significant dose–response associations between pain characteristics and incident frailty and prefrailty. Our findings indicated that high levels of pain-related interference in daily life, prolonged pain duration, and intense pain were strong, generic prognostic factors not only in primary pain care but also for future frailty. Pain characteristics, which are easily assessed using simple questions such as those used in the present study (adapted from the SF-12 Health Survey), can be used to stratify frailty risk. Therefore, primary care physicians should identify individuals with high-risk pain profiles and discuss appropriate pain management strategies with them. Multimodal interventions incorporating physical, psychological, and social therapies should be implemented depending on pain characteristics. Because frailty is a dynamic and potentially reversible condition, early and effective pain management is crucial for preventing the transition from prefrailty to frailty. Incorporating pain assessment into frailty screening is essential. Effective management of frailty should include appropriate pain treatment and vice versa. Notably, incorporation of comorbidities and self-rated health into the statistical models attenuated the observed associations, indicating a complex interplay that warrants further research. Nevertheless, chronic pain and pain-related interference in normal work remained strong predictors of frailty.

Although most associations in the present study between pain characteristics and frailty were not significantly affected by sex or age, a few sex-specific patterns emerged. The risk of prefrailty was higher in women than in men when pain interfered with normal work and mood. Male participants with moderate to severe pain–related interference in mood had a higher risk of frailty than did their female counterparts. Further studies are required to identify sex-based differences across populations with unique pain characteristics. Understanding these differences is particularly important in geriatric assessment because health outcomes are influenced not only by pain intensity but also by pain-related functional interference and sex-specific variations in pain perception and response. Recognizing these factors may help public-health policymakers devise robust strategies for healthy aging.

In summary, our findings provide robust evidence that can guide health-care professionals in identifying high-risk populations and implementing early management strategies for frailty prevention.

## Data Availability

The datasets presented in this article are not readily available because data are available from the applicants upon reasonable request and with permission of the Ministry of Health and Welfare in Taiwan. The data can be made available upon reasonable request from the Corresponding author. Requests to access the datasets should be directed to alexyeh@csmu.edu.tw.
