# Supplementary material for: Pain characteristics and incident frailty in community-dwelling older adults: dose–response relationships and sex differences in a longitudinal study on aging in Taiwan
Source: Front Public Health. 2026 Jul 31;14:1817545. doi: 10.3389/fpubh.2026.1817545 (PMC13475014; doi:10.3389/fpubh.2026.1817545)
Supplement: Supplementary file 1 [file Table_1.docx]

| **SUPPLEMENTARY TABLE S1** Baseline demographic, life-style and health characteristics of  included and excluded participants. | | | | | |
| --- | --- | --- | --- | --- | --- |
| **Variables** | **Included subjects**  **(N=5836)** | | **Excluded subjects**  **(N=1130)** | |  |
|  | **n** | **% (column) or**  **Mean±SD** | **n** | **% (column) or**  **Mean±SD** |  |
| **Sex** |  |  |  |  |  |
| Male | 2928 | 50.17% | 460 | 40.71% |  |
| Female | 2908 | 49.83% | 670 | 59.29% |  |
|  |  |  |  |  |  |
| **Age** |  |  |  |  |  |
| <65 | 3381 | 57.93% | 232 | 20.53% |  |
| 65-74 | 1561 | 26.75% | 278 | 24.60% |  |
| ≧75 | 894 | 15.32% | 620 | 54.87% |  |
|  |  |  |  |  |  |
| **Perceived financial situation** |  |  |  |  |  |
| Very dissatisfied, dissatisfied | 857 | 14.68% | 236 | 28.43% |  |
| Moderate | 2191 | 37.54% | 294 | 35.42% |  |
| Very satisfied, satisfied | 2788 | 47.77% | 300 | 36.14% |  |
|  |  |  |  |  |  |
| **Spouse** |  |  |  |  |  |
| No | 1391 | 23.83% | 571 | 50.58% |  |
| Yes | 4445 | 76.17% | 558 | 49.42% |  |
|  |  |  |  |  |  |
| **Education level** |  |  |  |  |  |
| Illiterate | 600 | 10.28% | 377 | 33.36% |  |
| Primary | 2143 | 36.72% | 459 | 40.62% |  |
| Junior / high school | 2130 | 36.50% | 235 | 20.80% |  |
| College degree or above | 963 | 16.50% | 59 | 5.22% |  |
|  |  |  |  |  |  |
| **Smoking habit** |  |  |  |  |  |
| No | 4870 | 83.45% | 1025 | 90.71% |  |
| Yes | 966 | 16.55% | 105 | 9.29% |  |
|  |  |  |  |  |  |
| **Alcohol consumption** |  |  |  |  |  |
| No | 3747 | 64.20% | 936 | 82.83% |  |
| Yes | 2089 | 35.80% | 194 | 17.17% |  |
|  |  |  |  |  |  |
| **Self-rated health** |  |  |  |  |  |
| Very poor, poor | 797 | 13.66% | 504 | 51.06% |  |
| Fair | 2382 | 40.82% | 330 | 33.43% |  |
| Very good, good | 2657 | 45.53% | 153 | 15.50% |  |
|  |  |  |  |  |  |
| **Numbers of chronic diseases** | 5836 | 1.04±1.11 | 1130 | 1.85±1.38 |  |
